# Pruritus as Reason for Encounter in General Practice

**DOI:** 10.4021/jocmr632w

**Published:** 2011-09-26

**Authors:** Thomas Frese, Kristin Herrmann, Hagen Sandholzer

**Affiliations:** a Department of Primary Care, Leipzig Medical School, Leipzig, Germany

## Abstract

**Background:**

Pruritus is a common reason for consulting the general practitioner. Data from a primary care setting have seldom been published. The goal of the recent investigation was to characterize the consultation prevalence of pruritus, frequency of diagnostic and therapeutic procedures, accompanying symptoms and results of encounter or diagnoses of patients with pruritus.

**Methods:**

Cross-sectional data were collected from randomly selected patients during the SESAM 2 study and compared with unpublished but publicly available data from the Dutch Transition Project and the published Australian BEACH study data.

**Results:**

Overall 64 of the 8,877 patients from the SESAM 2 study consulted a physician for pruritus. The male to female ratio was 1.0 : 1.3. Pruritus was more frequent in children and people aged over 75 years. Physical examination was performed in all patients. Further diagnostic measures were seldom necessary. Drugs were prescribed in 84% of the cases. Allergic contact eczema and infectious diseases of the skin were the most frequent results of encounter or diagnoses. Medical adverse effects and allergic reactions should be considered as causes of pruritus. We found no significant association to systemic diseases.

**Conclusions:**

In a primary care setting, pruritus occurs regularly. It is associated to (infectious) skin diseases. Acute dangerous courses are rare.

**Keywords:**

Pruritus; Itch; General practice; Primary care; Reason for encounter

## Introduction

Pruritus is a common problem in general practitioners’ consultations, the treatment of which has been an ongoing concern [1,2]. Pruritus, the Latin word for itch, is defined as the ”desire to scratch“. It is a distressing, subjective symptom that may interfere significantly with the quality of a patient's life [3]. It is known as a common symptom of dermatologic and systemic diseases [4]. Pruritus is often tormenting and therapy-resistant [4]. The pathophysiology of pruritus may differ depending on the underlying cause [5]. Pruritus is a complex process with etiology involving pain sensors, the autonomic nervous system, histamine and mediators such as serotonin, bradykinin, interleukin 2, prostaglandins, endorphins, streptokinase, opiates and emotional factors [6]. Itch is relieved by scratching, but the neural mechanisms that are responsible for this are poorly understood; spinothalamic tract neurons respond to itch-producing agents and transmit pruritic information to the brain. It has been observed that scratching the cutaneous receptive field of primate spinothalamic tract neurons produced inhibition during histamine-evoked activity [7]. Earlier investigations found that scratch-induced pain can abolish itch, and analgesic opioids can generate itch, which suggests an antagonistic interaction. A broad overlap between pain- and itch-related peripheral mediators and/or receptors was assumed [8].

Pruritus can be measured on different scales - e.g. visual analogue scale, ratio scale, generalized labelled magnitude scale. However, pruritus measurement is problematic, because of its subjective nature and poor localization [5]. Pruritus and itch can be classified aetiologically or clinically, depending on the clinical appearance of the patients, and are differentiated into disorders with or without primary or secondary skin lesions [9,10].

The frequency of pruritus in a primary care setting was assumed to be 8% [11]. A relatively high proportion (8.4%) of all adults in Oslo, Norway suffers from pruritus [12]. Approximately 1/3 of the patients with generalized pruritus of unclear origin have a systemic disorder [13] while the rest of the patients have an underlying skin disease [11]. The literature on pruritus as the primary reason for consulting the general practitioner is sparse.

The present study was planned to characterize the consultation prevalence, the management, the differential diagnoses and the significantly more frequent co-morbidities of patients encountering a typical primary care setting. The registration of the contacts for pruritus was a minor part of the study. Unpublished data from the Dutch Transition Project – another primary care study – were also analyzed with regard to pruritus as the reason for the encounter. The resulting description of the largest biggest data sample of patients presenting with pruritus was compared to the literature, especially the previously published BEACH-study.

## Materials and Methods

The Saxon Society of General Medicine (SGAM) contacted all general practitioners in Saxony by mail. They received no incentive for the participation. The study was set out to document reasons for encounter, diagnostic and therapeutic procedures as well as the result of encounter (chosen diagnosis).

Of the 2,510 physicians contacted, 270 general practitioners agreed to participate and 209 cooperated during the complete duration (one year). Cross-sectional data were collected from October 1, 1999 to September 30, 2000. Case recording was carried out on one day a week (Monday to Friday; either morning or afternoon consultation hours), chosen at random. Data were collected for one of ten patients previously known to the practitioner. Multiple recording of the same patient was avoided. House calls were not considered. A total of 8,877 patients were included.

A standardized data collection form was used [14]. It was developed by general practitioners (Leipzig Medical School and Saxon Society of General Medicine). The form was tested and evaluated during a pilot trial (SESAM 1). Each patient’s reasons for consulting, symptoms, diagnostic procedures, recent diagnoses and general morbidity were documented as well as therapeutic procedures. As far as possible, data were documented verbatim (according to the study instructions): either as told by the patients (e.g. reasons for consultation) or in the words of the physician (e.g. chronic diagnoses). Due to the random selection, the information was documented in a reasonably short time. Only completely filled-in forms were considered.

As described elsewhere, the SESAM 2 study provides independent and unbiased cross-sectional data from a typical primary care setting [15,16]. Because total morbidity was estimated there is no selection bias and the data can be assumed to be representative. The 1987 version of the International Classification of Primary Care (ICPC) was used to code the reasons for the encounter [17]. The SESAM 2 data was compared to those of two other investigations. Unpublished, but publicly available data from the Transition Project (described by Lamberts and Okkes [18]) were analyzed (total estimation of patients from about 20 Dutch general practitioners; 1985 till 2003). The data are available at www.transitieproject.nl. It can be analysed using the software that contains the database. The BEACH (Bettering the Evaluation and Care of Health) program, a continuous national study of general practice, began in April 1998 and is now in its eighth year. More than 7,500 general practitioners have participated and data are available for about 750,000 encounters [19,20]. Data from the BEACH study were published by Britt et al. [21].

Statistical analyses of the data were performed using Statistical Packages for Social Sciences (SPSS 15.0; SPSS Inc., Chicago, USA). As indicated, data was compared using Fisher’s exact test. Differences were stated as statistically significant for p < 0.05.

## Results

In the SESAM 2 study 8,877 patients, of whom 5,050 (56.9%) were females, 3,824 (43.1%) males and 3 were not specified, were reported from 209 general practitioners, and 13,632 reasons for consultation were coded.

Pruritus was ranked 44 of all reasons for consultation, and 64 (0.7%) of all patients suffered from pruritus, of whom 65.6% were female. The consultation prevalence was higher in children and adolescents, as well as in people aged over 75 years. The age distribution of the patients is shown in Table 1.

**Table 1:** Patient Distribution* (pd) on Different Age Groups and Consultation Prevalence** (cp) of Pruritus in Different Age Groups of the German SESAM 2 Study and the Dutch Transition Project Concerning the Condition of New or Previously Known Pruritus in a General Practice Setting

**Age (years)**	**SESAM 2 study** **(n = 64)**		**Transition Project initial consultation for new occurred pruritus** **(n = 5163)**		**Transition Project subsequent consultation for previously known pruritus** **(n = 1854)**	
	pd (%)	cp (%)	pd (%)	cp (%)	pd (%)	cp (%)
0 to 4	3.1	1.8	4.9	2.6	4.5	0.9
5 to 14	7.8	1.7	9.3	3.3	7.1	0.9
15 to 24	6.3	0.4	11.6	3.7	8.2	0.9
25 to 44	15.6	0.5	32.0	3.6	29.4	1.2
45 to 64	34.4	0.8	21.1	3.3	20.1	1.1
65 to 74	14.1	0.5	11.5	3.7	16.3	1.9
> 75	18.8	1.0	9.6	3.4	14.4	1.8

*Patient distribution (pd) is the percentage of the specified age group of the patients with pruritus (e.g. 7.8% of all SESAM 2-patients with pruritus were 5 to 14 years old); **Consultation prevalence (cp) is the percentage of patients with pruritus in the age group related to all patients of the age group (e.g. among SESAM 2-children from 5 to 14 years of age 1.7% encounter for pruritus).

All patients with pruritus underwent a physical examination (Table 2). The prescription of drugs was the most frequently performed physician’s action (Table 3). Most of the patients had skin symptoms such as erythemas, redness and insect bites. Results of encounter or diagnoses with significant association to pruritus are given in Table 4.

**Table 2:** Physician’s Action (%) in the SESAM 2 Study and the Transition Project Concerning the Condition of New or Already Known Pruritus. Procedures That Are Not Explicit Diagnostic Were Also Registered in Table 3

**Physician’s action**	**SESAM 2 study** **(n = 64)**	**Transition Project initial consultation for new occurred pruritus** **(n = 5163)**	**Transition Project subsequent consultation for previously known pruritus** **(n = 1854)**
Physical examination	100.0	92.7	86.0
Follow up consultation	75.0	N/A	N/A
Laboratory investigations	15.6	4.1	6.6
Referral	14.1	0.05 (pc*)	0.2 (pc*)
Other diagnostics	6.3	0.35	0.7
ECG	3.1	N/A	N/A
Hospitalization	1.6	1.6 (sc**)	9.1 (sc**)
Excision/histology	0	0.3	0.3

*pc: primary care; **sc: specialized care.

**Table 3:** Physician’s Action (%) in the SESAM 2 Study and the Transition Project Concerning the Condition of New or Already Known Pruritus. Procedures That Are Not Explicitly Therapeutic Were Also Registered in Table 2

**Physician’s action**	**SESAM 2 study** **(n = 64)**	**Transition Project initial consultation for new occurred pruritus** **(n = 5163)**	**Transition Project subsequent consultation for previously known pruritus** **(n = 1854)**
Drug prescription	84.4	84.3	79.0
Follow up consultation	75.0	N/A	N/A
Other therapy	20.3	0.6	1.0
Physicians advice	10.9	20.1	19.3
Referral	14.1	0.05 (pc*)	0.2 (pc*)
Incapacity to work	3.1	N/A	N/A
Hospitalization	1.6	1.6 (sc**)	9.1 (sc**)

*pc: primary care; **sc: specialized care.

**Table 4:** Comparing the Incidence (Number (N) and Percentage (%)) of Results of Encounter or Diagnoses in General Practice Patients With Pruritus to Those Without Pruritus (SESAM 2 Study) Shows That Pruritus is Significantly Associated to Skin Diseases

Diagnosis*	**Pruritus**		**Without Pruritus**		**p-value** **(Fisher)**
	**n**	**%**	**n**	**%**	
Contact dermatitis	15	23.4	49	0.6	0.000
Dermatophytosis**	5	7.8	25	0.3	0.000
Herpes Zoster	5	7.8	21	0.2	0.000
Allergy/allergic reaction	4	6.3	10	0.1	0.000
Urticaria***	3	4.7	5	0.1	0.000
Scabies/Pediculosis	3	4.7	4	0	0.000
Varicella	3	4.7	8	0.1	0.000
Skin injury other	3	4.7	96	1.1	0.034
Atopic dermatitis	2	3.1	4	0	0.001
Atherosclerosis/peripheral vascular disease	2	3.1	10	0.1	0.003
Pruritus (not explained)	1	1.6	1	0	0.014
Adverse effect medical	1	1.6	1	0	0.014
Exanthema	1	1.6	1	0	0.014
Risk factor	1	1.6	2	0	0.021
Other viral exanthem	1	1.6	5	0.1	0.042
Perianal itching	1	1.6	1	0	0.014
Phobia/compulsive disorder	1	1.6	3	0	0.029
Seborrheic dermatitis	0	0	9	0.1	N/A
Psoriasis	0	0	8	0.1	N/A
Sun burn	0	0	15	0.2	N/A

*Further diagnoses of patients with pruritus omitted from the table because of lacking significance: Other infectious disease, gastrointestinal infection, anal fissure/perianal abscess, otitis externa, postural hypotension, neck symptom/complaint, fracture: hand/foot bone, bursitis/tendinitis/synovitis, tennis elbow, other musculoskeletal disease, Vertigo/dizziness, upper respiratory infection acute, Acute bronchitis/bronchiolitis, warts, Skin infection other, and cystitis/urinary infection. **Dermatophytosis: A superficial fungus infection involving the stratum corneum of the skin, hair, and nails; ***Urticaria: A pruritic skin eruption characterized by transient wheals of varying shapes and sizes with well-defined erythematous margins and pale centers.

The most common chronic diseases among pruritus patients in the SESAM study were arterial hypertension, osteoarthritis, back pain, coronary heart disease and diabetes. None of them showed a significant association with pruritus. Only dermatologic diseases were significantly associated with pruritus (Table 4). Skin diseases were diagnosed in about 50% of the patients with pruritus; infectious diseases - e.g. herpes zoster, varicella and mycoses - were the second dominating group. The most frequent skin disease was allergic contact eczema. Table 5 summarizes the consultation prevalence of different skin diseases in patients with pruritus.

**Table 5:** Frequency (%) of the Most Frequent Results of Encounter or Diagnoses for Primary Care Patients With a Complaint of Pruritus

**Diagnosis**	**SESAM 2 study** **(n=64)**	**Transition Project initial consultation for new occurred pruritus** **(n = 5163)**	**Transition Project subsequent consultation for previously known pruritus** **(n=1854)**	**BEACH study** **(n=2971) [16]**
Contact dermatitis	26.6	24.4	27.8	30.7
Dermatophytosis	7.8	11.5	5.2	5.8
Atopic dermatitis, eczema	7.8	5.4	10.8	1.3
Herpes Zoster	7.8	0.8	0	0
Urticaria	4.7	9.9	6.3	8.0
Scabies, pediculosis	4.7	1.0	0.9	4.8
Varicella	4.7	0.3	0.1	0
Adverse effect medical	6.3	1.9	1.1	2.0
Pruritus (not explained)	1.6	20.0	22.6	11.6
Seborrheic dermatitis	1.6	3.5	5.0	5.5
Psoriasis	1.6	0.5	1.9	2.3
Allergy, allergic reaction	1.6	1.8	1.4	4.7
Other skin disease	3.1	2.3	2.4	3.5
Insect bite	0	0.9	0.2	2.6
Solar keratosis, sunburn	0	0	0	2.3

For the Dutch Transition Project a total of 149,238 patients were examined over the period 1985 to 2003. Of these patients 7,017 were coded as giving pruritus as the reason for the consultation. The majority (5,163 of these 7,017) were coded for the first time with pruritus, and 1,854 patients had known pruritus. The proportion of patients with pruritus was also small in The Netherlands (0.5% of all registered patients). Pruritus was most commonly associated with a skin disease (91%). The general practitioners physically examined 44% of the affected patients, 40% were prescribed medication. At least 253 patients were referred to a specialist, more than 90% of these to a dermatologist.

## Discussion

Pruritus is a pressing problem that is often underestimated in clinical research studies. It is a common reason for consulting the general practitioner. However, few detailed studies in a primary care setting have been performed. Data were reported for only a single study [21]. In spite of methodological differences; the consultation prevalence of pruritus is about 0.5% in the three studies considered (SESAM 2, BEACH study, Transition Project). This is much lower than stated by others [11] and also lower than the population prevalence as reported by Dalgard et al [12]. Skin diseases and itch are more common among the elderly because of the chronically sun-damaged and more vulnerable skin [22,23]. The male to female ratio was reported to be 1.0 : 1.2 [24] to 1.0 : 1.5 [21]; we found a ratio of 1.0 : 1.3. Our findings do strongly suggest that pruritus is an accompanying symptom of skin diseases (Table 3). This was confirmed by others [21]. It could be mentioned that the estimated data on the management of pruritus may be influenced by the data estimation itself (when physicians record their actions in the consulting room, especially actions like physical examination may be high due to socially desirable behavior). As stated above, in the SESAM 2 study we estimated all reasons for encounter, not only pruritus. Thereby the procedures are comparable between different reasons of encounter within the SESAM 2 study and within the Transition Project.

The referral rates differ markedly between the German SESAM 2 study (14.1%), the Dutch Transition Project (0.05%) and the Australian BEACH study (4.5%). This might be caused by differences in the health care systems and differences in the study design. Also the referrals in the SESAM 2 study might have been arranged for reasons other than pruritus. It is remarkable that patients with known pruritus were frequently referred (Table 2). The management of most patients with skin diseases does not require specialized care [24]. Drug prescription is the most frequent (about 84%) therapeutic physician’s action [21] (Table 3). In particular, acute and localized forms of pruritus are most likely treated with topical steroids [21].

Pruritus may be a side effect of systemic diseases or drug therapies [25,26]. Unfortunately, there was no sufficient information about the medication of the included patients. Pruritus as a side effect of drug treatment was documented in about 1.5% of pruritus patients in the Transition Project as well as the SESAM 2 study (Table 5). We were not able to investigate pruritus as a side effect of drug therapy in the recent setting due to the low number of pruritus cases in the sample. In a specialized setting (university department of dermatology) pruritus was the initial symptom of a not yet diagnosed disease in 7 of 50 patients [13]. The underlying diseases included hypothyroidism, gastric adenocarcinoma, hepatitis C, HIV, laryngeal carcinoma, graft-versus-host disease, and chronic lymphocytic leukemia [13]. Analysing data from the Transition Project revealed that this was not the cause in a (low prevalence) primary care setting. It should be borne in mind that two recent studies (SESAM 2, BEACH study) differentiated neither between local and generalized nor between acute and chronic pruritus. Under the condition of prolonged generalized and unexplained itch, systemic diseases should be taken into account as differential diagnoses. In the recent studies, the rate of unexplained itch ranged from about 2 to 20% (Table 4); in a specialised care setting it was 6% [24]. It is conclusive that pruritus caused by a systemic disease may not regularly occur in general practitioners’ consultation.

None of the recent studies provided sufficient information about the frequency of patients with pruritus lacking pathological skin signs (*pruritus sine materia*). Pruritus as a symptom is often not recorded in studies or is ignored in disorders that are associated with pruritus or in which it occurs as one of several symptoms. Therefore data are only available for the individual clinical pictures [4]. A recent paper from the Netherlands showed that itch and pain frequently occur, often in combination, in patients with dermatological diseases, and that women are affected particularly often [27]. Overall, it has been possible to confirm that the most common cause is a dermatological disease, regardless of whether this is local or generalized pruritus. The use of classification systems for (new) pruritus appears to be unnecessary in a primary care setting.

In conclusion, pruritus is regularly a reason for consulting the general practitioner. Although a lot of systemic diseases may cause pruritus, most of the primary care patients with pruritus do have a dermatological problem and systemic causes do not occur regularly. Referring patients for further diagnosis is only necessary in a small number of the cases. In no patients was the course of the disease acutely dangerous. Classification systems for pruritus are of minor importance in a primary care setting. Patients with persistent generalised pruritus of unknown origin should be further diagnosed with focus on systemic diseases.

## References

[1] Wawersig R (1956). Management of pruritus in general practice with ilvin. Munch Med Wochenschr.

[2] Forman L (1954). Pruritus and its management. Br Med J.

[3] Hiramanek N (2004). Itch: a symptom of occult disease. Aust Fam Physician.

[4] Stander S, Weisshaar E, Steinhof M, Luger TA, Metze D (2003). Pruritus--pathophysiology, clinical features and therapy--an overview. J Dtsch Dermatol Ges.

[5] Langner MD, Maibach HI (2009). Pruritus measurement and treatment. Clin Exp Dermatol.

[6] Streit M, Von Felbert, Braathen LR (2002). Pruritus sine marteria. Pathophysiology, diagnostic assessment and therapy. Hautarzt.

[7] Davidson S, Zhang X, Khasabov SG, Simone DA, Giesler GJ, Jr (2009). Relief of itch by scratching: state-dependent inhibition of primate spinothalamic tract neurons. Nat Neurosci.

[8] Ikoma A, Steinhoff M, Stander S, Yosipovitch G, Schmelz M (2006). The neurobiology of itch. Nat Rev Neurosci.

[9] Stander S, Weisshaar E, Mettang T, Szepietowski JC, Carstens E, Ikoma A, Bergasa NV (2007). Clinical classification of itch: a position paper of the International Forum for the Study of Itch. Acta Derm Venereol.

[10] Stander S, Weisshaar E, Mettang T, Streit M, Darsow U, Schneider G, Metze D (2006). Clinical classification of chronic pruritus. Interdisciplinary consensus proposal for a diagnostic algorithm. Hautarzt.

[11] Kantor GR, Lookingbill DP (1983). Generalized pruritus and systemic disease. J Am Acad Dermatol.

[12] Dalgard F, Svensson A, Holm JO, Sundby J (2004). Self-reported skin morbidity in Oslo. Associations with sociodemographic factors among adults in a cross-sectional study. Br J Dermatol.

[13] Zirwas MJ, Seraly MP (2001). Pruritus of unknown origin: a retrospective study. J Am Acad Dermatol.

[14] Wockenfu R (2010). berprfung der Reliabilitt der ICD-10 in der Allgemeinmedizin. Leipzig Medical School.

[15] Wockenfuss R, Frese T, Herrmann K, Claussnitzer M, Sandholzer H (2009). Three- and four-digit ICD-10 is not a reliable classification system in primary care. Scand J Prim Health Care.

[16] Frese T, Sandholzer H, Voigt S, Voigt R (2008). Epidemiology of diabetes mellitus in German general practitioners' consultation--results of the SESAM 2-study. Exp Clin Endocrinol Diabetes.

[17] Soler JK, Okkes I, Wood M, Lamberts H (2008). The coming of age of ICPC: celebrating the 21st birthday of the International Classification of Primary Care. Fam Pract.

[18] Lamberts H, Okkes I (1999). Patients with chronic alcohol abuse in Dutch family practices. Alcohol Alcohol.

[19] Britt HC, Miller GC (2000). The BEACH study of general practice. Med J Aust.

[20] Britt H (2003). BEACH--bettering the evaluation and care of health: a continuous national study of general practice activity. Commun Dis Intell.

[21] Britt H, Pan Y, Miller GC, Valenti L, Charles J, Knox S, Henderson J (2004). Presentations of 'itch' in Australian general practice. Aust Fam Physician.

[22] Wheeler T (2009). Managing pruritus in the older person. Br J Community Nurs.

[23] Webster GF (2001). Common skin disorders in the elderly. Clin Cornerstone.

[24] Symvoulakis EK, Krasagakis K, Komninos ID, Kastrinakis I, Lyronis I, Philalithis A, Tosca AD (2006). Primary care and pattern of skin diseases in a Mediterranean island. BMC Fam Pract.

[25] Weisshaar E, Kucenic MJ, Fleischer AB, Jr (2003). Pruritus: a review. Acta Derm Venereol Suppl (Stockh).

[26] van der Linden PD, van der Lei J, Vlug AE, Stricker BH (1998). Skin reactions to antibacterial agents in general practice. J Clin Epidemiol.

[27] Verhoeven EW, Kraaimaat FW, van de Kerkhof PC, van Weel C, Duller P, van der Valk PG, van den Hoogen HJ (2007). Prevalence of physical symptoms of itch, pain and fatigue in patients with skin diseases in general practice. Br J Dermatol.

